# Admixture and Gene Flow from Russia in the Recovering Northern European Brown Bear (*Ursus arctos*)

**DOI:** 10.1371/journal.pone.0097558

**Published:** 2014-05-19

**Authors:** Alexander Kopatz, Hans Geir Eiken, Jouni Aspi, Ilpo Kojola, Camilla Tobiassen, Konstantin F. Tirronen, Pjotr I. Danilov, Snorre B. Hagen

**Affiliations:** 1 Bioforsk - Norwegian Institute for Agricultural and Environmental Research, Svanvik, Norway; 2 Department of Biology, University of Oulu, Oulu, Finland; 3 Finnish Game and Fisheries Research Institute, Oulu, Finland; 4 Institute of Biology, Karelian Research Centre of the Russian Academy of Science, Petrozavodsk, Russia; University of Idaho, United States of America

## Abstract

Large carnivores were persecuted to near extinction during the last centuries, but have now recovered in some countries. It has been proposed earlier that the recovery of the Northern European brown bear is supported by migration from Russia. We tested this hypothesis by obtaining for the first time continuous sampling of the whole Finnish bear population, which is located centrally between the Russian and Scandinavian bear populations. The Finnish population is assumed to experience high gene flow from Russian Karelia. If so, no or a low degree of genetic differentiation between Finnish and Russian bears could be expected. We have genotyped bears extensively from all over Finland using 12 validated microsatellite markers and compared their genetic composition to bears from Russian Karelia, Sweden, and Norway. Our fine masked investigation identified two overlapping genetic clusters structured by isolation-by-distance in Finland (pairwise *F_ST_* = 0.025). One cluster included Russian bears, and migration analyses showed a high number of migrants from Russia into Finland, providing evidence of eastern gene flow as an important driver during recovery. In comparison, both clusters excluded bears from Sweden and Norway, and we found no migrants from Finland in either country, indicating that eastern gene flow was probably not important for the population recovery in Scandinavia. Our analyses on different spatial scales suggest a continuous bear population in Finland and Russian Karelia, separated from Scandinavia.

## Introduction

Habitat fragmentation and anthropogenic disturbance is a global threat to wildlife, with impacts such as declining population sizes and reduced gene flow among populations. Both effects are widely reported to promote genetic drift and oppose long-term population viability [1]. Certain species are particularly sensitive to loss of inter-population connectivity [2]–[4]. Examples are many apex predators, characterized by small population size, long generation times, large home ranges, and high levels of human persecution [2], [5]–[8].

During the last century, large terrestrial carnivores declined both in numbers and geographic distribution (see e.g. [4], [9]). Even though they were almost extirpated in most of Europe, large carnivores have now recovered in some areas and populations are expanding [2], [4], [9], [10]. An important step towards understanding the underlying causes of recovery is to determine the current degree of gene flow and genetic differentiation among large carnivore populations across national borders [10].

In Finland, the brown bear (*Ursus arctos*) was distributed throughout the country until the beginning of the 19th century [11]. At the end of the 19th century, bears seemed to be extinct from central, southern, and western Finland, while observations of bears were still reported in the north and east [12]. Historic records indicate that the brown bear population of Finland went through a demographic bottleneck, with at least 9,000 individuals killed between 1875 and 2000 [12]. It is assumed that the population size reached its minimum between 1920 and 1950. In 1963, the remaining number of bears was estimated to be about 150 individuals [13]. Estimates based on bear observations suggested an increase from approximately 300 to 800 individuals between 1978 and 2003 [14], [15]. Migration from Russia into Finland has been assumed to have supported the growth of the Finnish population [11], [13], [16]–[18]. The most recent estimates based on observations of the number of litters-of-the-year are suggestive of a number between 1,150 and 1,950 bears in 2009, with highest densities in the south along the Finnish-Russian border [19]. In this area, records of killed bears also indicate a particularly high proportion of female bears [16], [20], [21].

The Finnish brown bear population is located centrally between the populations of Russia and Scandinavia. The Republic of Karelia and the Murmansk Oblast in Russia are the neighboring districts towards Finland. Based on hunting records, observations, and track counting the estimated numbers of bears in these districts in 1990 were about 3,500 and 500 bears, respectively [22], [23]. Towards the north, Finland shares the border with Norway, where noninvasive genetic sampling of scats and hairs has documented small brown bear populations in the Pasvik Valley, in the Karasjok-Anarjohka region, and in the area of Dividalen in Troms [24], [25]. In Sweden, towards the west, effort-corrected moose-hunter observations combined with noninvasive genetic capture-recapture studies have been used to estimate the population to be approximately 3,300 bears [26].

Recent studies of brown bears from Northern Europe suggest both genetic structuring due to isolation-by-distance (IBD) and the existence of separate genetic populations [24], [25], [27]–[31]. Previously, we have detected bi-directional migration rates of about 30% between bears in Eastern Finland and bears further east in Arkhangelsk, Russia [28]. Another study applying autosomal microsatellites to a restricted number of samples suggested that Finnish bears are divided into a northern and a southern subpopulation [30]. A recent mitochondrial genome study of bears in Northeastern Europe also indicated a northern and a southern cluster influenced by mitogenetic haplogroups from European Russia [32]. Furthermore, we have found indications that the connectivity between the bear populations in Eastern Finland and Scandinavia to be limited [25].

Since the recovery of brown bears in Finland is assumed to be explained by high gene flow from Russian Karelia, one should expect a low degree of genetic differentiation between brown bears from these areas today, which has not been sufficiently tested. In addition, the results of the previous studies suggesting more than one subpopulation of bears in Finland [30], may be inaccurate because of IBD and selective sampling. In contrast, in this study we have sampled individuals extensively and continuously all over Finland to answer the question whether or not there is a northern and southern population of bears in Finland. We included samples from Russia to scrutinize the influence of eastern gene flow on the composition of the Finnish bear population. In a last step, we included our previously published genetic data on bears from Scandinavia (Sweden and Norway) to investigate the connectivity further westwards. Comparing results on the genetic structure from three different geographic scales allowed us to determine more precisely the underlying genetic admixture and gene flow in Northwestern Europe.

## Materials and Methods

### Sampling

All samples were collected from dead animals, harvested legally in Finland and Russia. Legal harvest of bears in Finland in the different hunting districts follows an annual quota corresponding to the estimated abundance and distribution of brown bears in those areas [19], and the sampling in this study follows this distribution throughout Finland. Tissue samples were obtained by our collaborators namely the Finnish Game and Fisheries Research Institute and the Karelian Research Centre of the Russian Academy of Science. No ethic permit was required, as the sample collection did not involve live animals.

**Figure 1:**
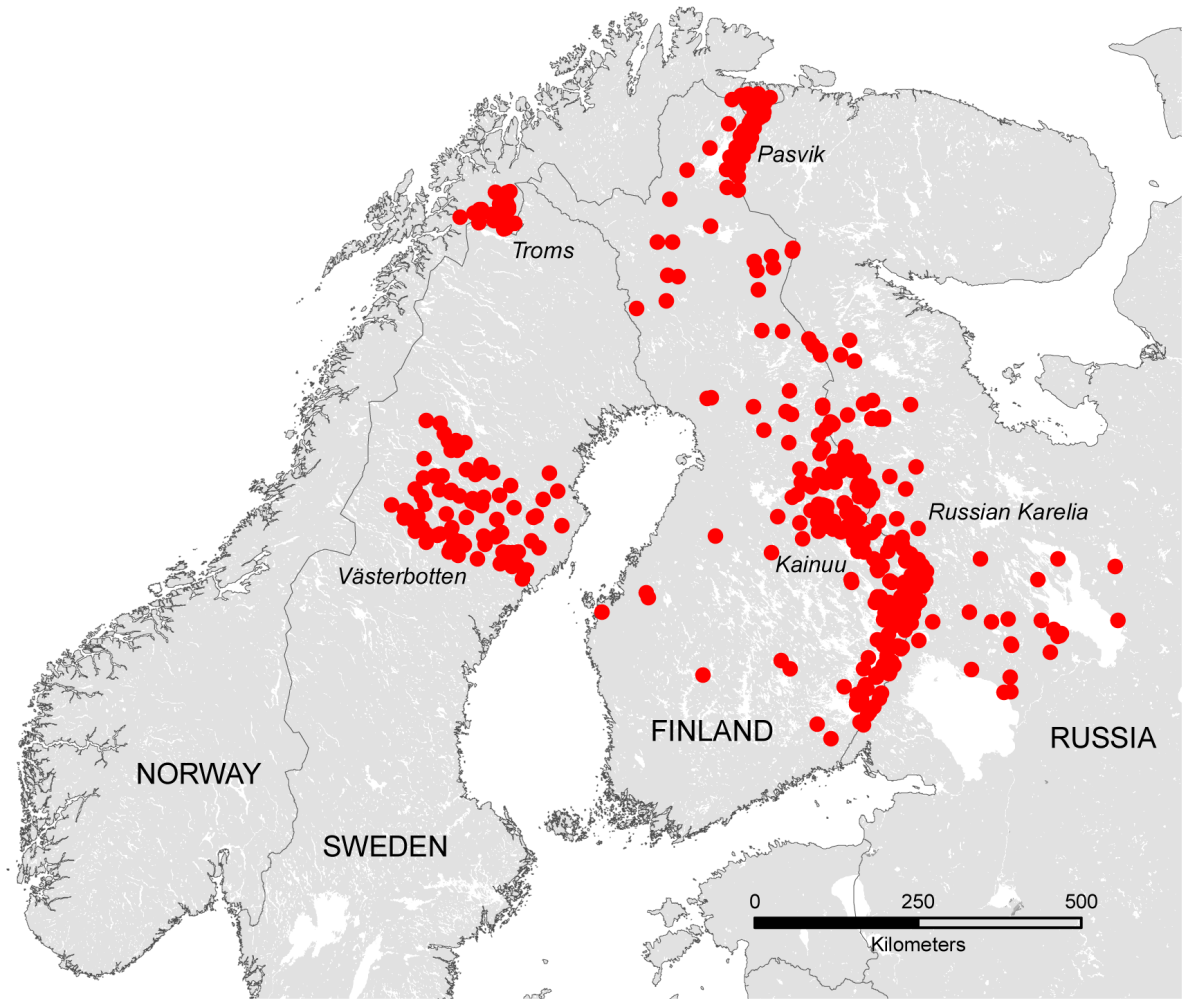
Sampling locations of the brown bears (*N* = 517) in Northern Europe. Samples were collected from 2006 to 2010 and each individual is represented by a red dot.

In our study, we analyzed the data of a total of 517 bears from 2006 to 2010 (Figure 1, Table S1), including 286 tissue samples from individuals from Finland, collected annually from legally harvested bears (91 females, 195 males). To investigate gene flow from Russia into Finland and westwards to Scandinavia, we included previously genotyped individuals from Norway (*N* = 97), Sweden (*N* = 84) and Russia (*N* = 22); (see Kopatz et al. 2012 and Schregel et al. 2012) and 28 additional tissue samples from Russian Karelia (total *N* = 50 bears) from the same time period.

### Molecular Analysis

Immediately after collection, tissue samples were stored in 95% ethanol until extraction. Samples were extracted with DNeasy Tissue Kit (Qiagen), following the manufacturer’s instructions, and genotyped using 12 different dinucleotide markers (short-tandem-repeats, STRs) developed for bears: G1A, G1D, G10B, G10L [33], [34]; Mu05, Mu09, Mu10, Mu15, Mu23, Mu50, Mu51 and Mu59 [35]. We have previously validated these STRs for their species sensitivity, precision and probability of identity [24], [27]. The protocol for PCR and fragment analysis can be found in Andreassen et al. [27]. Our laboratory procedures follow the guidelines for the analysis of non-human forensic DNA material [36]. We verified the uniqueness of all genotypes by calculating their probability of identity using the software Gimlet version 1.3.3 [37]. Genotypes were tested in Micro-Checker version 2.2.3 for possible allelic dropout, presence of null alleles, and scoring errors caused by stutter peaks [38].

### Population Structure

We tested for genetic structure using two Bayesian assignment algorithms (Structure and Geneland) and factorial correspondence analysis (FCA). Since earlier studies have indicated a restricted number of genetic clusters in Northwestern Europe [25], [28]–[30], we set our Bayesian analyses on genetic clustering to a maximum of *K* = 10. In Structure version 2.3.3 [39], [40], we assumed population admixture and correlated allele frequencies within the population. Ten independent runs for each *K* value between one and ten were performed. For each run, we set a burn-in period of 100,000 Markov Chain Monte Carlo (MCMC) iterations, followed by sampling of 1,000,000 iterations. The results were post-processed with the ad-hoc approach of Evanno et al. [41] to estimate the number of genetic clusters using Structure Harvester [42]. A membership coefficient (*q*) above 0.6 has been considered as a feasible cut-off membership value to assign individuals to a population with confidence, since more than 50% of the genome is assigned to a group and therefore suggests inferred ancestry [43], [44]. Previous studies on bears have used a membership coefficient (*q*) of 0.7 [30], [44]. Thus, we have applied a threshold value of *q*>0.7 in this study.

In Geneland [45], we ran five independent runs, where the parameters for possible populations were *K* = 1 to 10, and the number of MCMC iterations was 1,000,000, with a thinning of 100. The maximum rate of Poisson process was set to 100, and the maximum number of nuclei was 300. Geographical location of the samples (longitude, latitude) was included into the analysis. FCA was performed with the program Genetix 4.05.2 [46].

To determine the degree of differentiation among genetic clusters, AMOVA analyses and pairwise *F_ST_* values were calculated with the program Arlequin version 3.5.1.2 [47].

### Isolation-by-distance

We calculated IBD among pairs of brown bears in Finland and Russian Karelia using the software Spagedi version 1.3 [48] with the kinship coefficient by Loiselle et al. [49].

### Gene Flow

To further test the east-west gene flow hypothesis we estimated the amount of migration between the bears in Finland and Russian Karelia as well as between Finland and Scandinavia using two different methods. Firstly, the effective number of migrants (*Nm*) was estimated using the private allele method [50] implemented in the program Genepop [51]. Secondly, to identify possible recent migrants, we estimated the likelihood of a bear to belong to the population it was sampled using the individual Bayesian assignment method in the program Geneclass 2 [52]. We used the algorithm by Rannala and Mountain [53] and resampling as described in Paetkau et al. [54] to identify first generation migrants. The simulation was set to 10,000 individuals and the type I error (alpha) to 0.05.

### Genetic Diversity

We calculated number of alleles, expected and observed heterozygosity with the program Arlequin version 3.5.1.2 [47]. Inbreeding coefficients and tests for linkage disequilibrium between pairs of loci were performed with the program Genetix 4.05.2 [46] using the method by Black and Krafsur [55]. Deviations from Hardy-Weinberg equilibrium (HWE) were tested with Fisher’s method [56] for all loci and populations with the program Genepop version 4.0 [51], with unbiased *P* values by a Markov chain method of 1000 burn-in iterations, 500 batches and 1000 iterations per batch.

### Population Bottlenecks

For the bears from Finland and Russia, we tested for larger observed heterozygosity than expected to detect possible genetic bottlenecks in the recent history of the bear populations with the program Bottleneck 1.2.02 [57]. We applied the two-phase mutation model using 95% single step mutations to estimate the expected heterozygosities (20,000 iterations) and tested the significance of the differences between observed and expected heterozygosities using the Wilcoxon test. Further, we applied the M ratio test to investigate if there are signs of genetic bottlenecks further in the past (>100 generations) and therefore we calculated the modified Garza-Williamson indices [58] for the clusters found implemented in the program Arlequin version 3.5.1.2 [47].

## Results

### Population Structure in Finland and Russian Karelia

**Figure 2:**
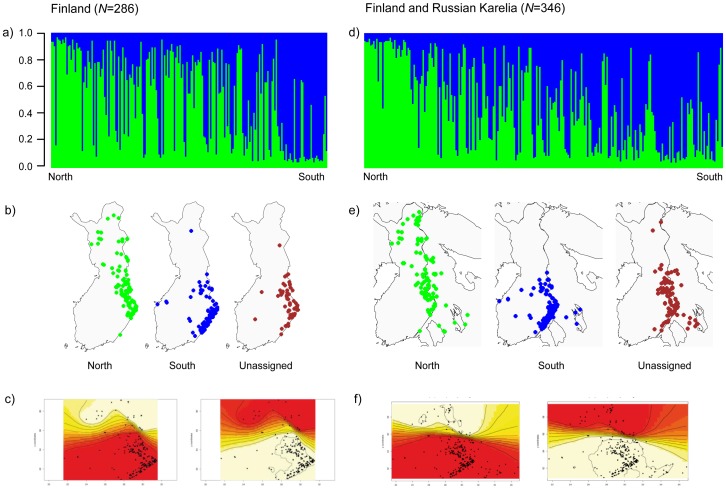
Bayesian clustering results of Finnish and Russian Karelian bears with Structure (Pritchard et al. 2000). Bar plots show the assignment probabilities for each bear to one of the identified two clusters when only samples from Finland were analyzed (a) and samples from Finland and Russian Karelia pooled together (d); northern cluster (green), southern cluster (blue). The y-axis shows the calculated membership coefficient (*q*). Individuals are arranged by latitude from north (left) to south (right). (b and e) The maps show the genotypes in accordance to their assignment in Structure and geographical location. Individuals which were not assigned unambiguously (membership coefficient *q*<0.7) are shown on a separate map as dark red dots. (c and f) Maps on the bottom show the assignment with the program Geneland (Guillot et al. 2005).

**Figure 3:**
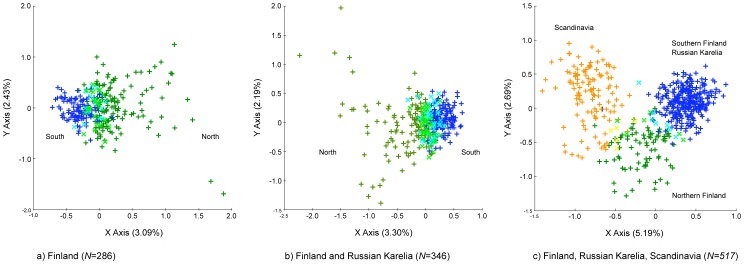
Factorial correspondence plots for brown bears sampled 2006–2010 in Finland, Russia, Norway and Sweden. Different colors represent the clusters identified by the Bayesian clustering approach. (a) FCA analysis of the Finnish samples only: northern cluster (green), northern cluster with a membership coefficient (*q*) <0.7 (light green), southern cluster (blue), southern cluster with assignment membership probability <0.7 (cyan). (b) FCA analysis of Finnish and Russian Karelian samples: northern cluster (green), northern cluster with an assignment membership probability <0.7 (light green), southern cluster (blue), southern cluster with assignment membership probability <0.7 (cyan). (c) FCA analysis of Finnish, Russian Karelian populations and bears sampled in Northern Norway and Sweden: northern cluster (green), northern cluster with a membership coefficient <0.7 (light green), southern cluster (blue), southern cluster with assignment membership probability <0.7 (cyan), western (Scandinavian) cluster (orange), western cluster with an assignment membership probability <0.7 (yellow).

The Structure clustering approach suggested two genetic clusters in Finland with a high degree of admixture and geographical overlap (Figure 2a and b, Figure S1a, and Figure S2a). While one cluster was spread almost throughout two-thirds of the country, the other one was restricted to the southern part of Finland (Figure 2b). A total of 60 bears (21%) were not assigned unambiguously to the identified clusters (membership coefficient *q*<0.7), and those were mainly found in the zone where the clusters overlapped (Figure 2b). All alternative models of population structure using a larger number of clusters (*K* = 3 to 5) had lower likelihoods and showed substantially higher numbers of unassigned individuals up to 72% (Figure S2a). Geneland identified also two clusters, i.e. a northern and a southern one (Figure 2c). FCA supported the results by Structure, showing two, overlapping groups of bears (Figure 3a). Bears not unambiguously assigned by Structure showed highest similarity and occurred on the FCA plot between the two clusters (Figure 3a).

Similar to the results in Finland alone, assigning genotypes from Finland and Russian Karelia together also suggested two genetic clusters (Figure 2d, Figure S1b and Figure S2b). One cluster spread throughout the distribution range while the other one was concentrated mainly to the southern part of the study region (Figure 2e). Admixture and geographical overlap as suggested by the assignment probabilities (Figure 2d) could be as well observed here (Figure 2e). Similarly, most of the unambiguously assigned genotypes, 79 individuals in total (22.8%), were found in the geographic overlap zone (Figure 2e). Geneland showed two genetic clusters: a northern and southern cluster, with a distinctive border in the middle of Finland (Figure 2f). FCA analyses supported the results by Structure of two, overlapping genetic groups (Figure 3b). Repeatedly, bears which were not assigned unambiguously seem to highlight an admixture group between the two identified clusters (Figure 3b).

### Connectivity with Scandinavia

**Figure 4:**
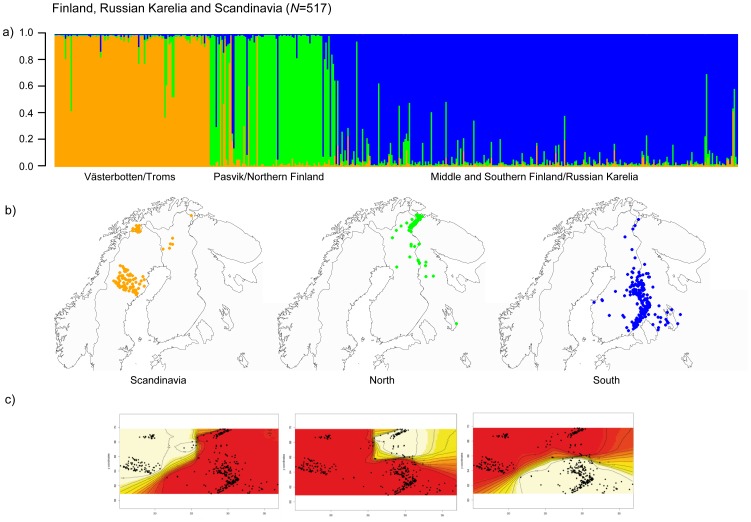
Assignment of bears sampled 2006–2010 in Northwestern Europe with the program Structure (Pritchard et al. 2000). Bar plots show the assignment probabilities for each bear to one of the three identified clusters (a). Genotypes are sorted “clockwise” in accordance to their location from south-west in Sweden to south east in Finland and Russia according to from left to right: in orange Västerbotten (south-north) and Troms (west-east), in green Northern Finland (west-east) and Pasvik (north-south), in blue Southern Finland and Russian Karelia (north-south). The y-axis shows the calculated membership coefficient (*q*). (b) The maps show the genotypes in accordance to their assignment in Structure and geographical location: western cluster in Scandinavia, namely Västerbotten and Troms, shown in orange; northern cluster in green and southern cluster in blue. (c) Maps on the bottom show the assignment with the program Geneland (Guillot et al. 2005).

After pooling Finnish and Russian Karelian bears together with bears from the northern trans-border area of Pasvik in Norway and Russia, Troms in Norway, and Västerbotten in Sweden, the Bayesian clustering approaches (Structure and Geneland) suggested three genetic clusters: a western one, including mainly genotypes from Scandinavia, namely Västerbotten and Troms, a northern one, including genotypes from Pasvik and northern Finland and Murmansk, and a southern cluster containing genotypes from middle and southern Finland as well as Russian Karelia (Figure 4a and b, Figure S1c and Figure S2c). In comparison to the analyses of population structure using samples from Finland and Russian Karelia only, the border between the two groups in Finland and Karelia was located a bit further north (Figures 2 and 4). Here, we found approximately 20 individuals in the north assigned to the southern cluster, compared to the results when using solely Finnish bear samples, where only one individual in the north has been assigned to the southern cluster. Furthermore, only 18 (3.5%) of the individuals could not be assigned unambiguously, suggesting that most of the unassigned genotypes found at smaller spatial scales were intermediate genotypes from the admixture zone rather than individuals from an unknown population. Genotype assignment with Structure in accordance to the sampling location is shown in Figure 4b. FCA analysis was in line with the results by the Bayesian assignments and visualized three distinctive groups of bears (Figure 3c).

### Population Differentiation in Finland and Russian Karelia

The pairwise *F_ST_* values between the two subpopulations were significant (*P*<0.001), with *F_ST_* = 0.025 between the northern and southern cluster found in Finland and *F_ST_* = 0.026 between the two clusters identified when Finnish and Russian samples were analyzed together. AMOVA analysis within Finland showed that 2.54% of the variation was between the clusters and 97.46% within them (*P*<0.01). For the north-south division in Finland and Russian Karelia, 2.59% variation was between the clusters and 97.41% variation within the them, respectively (*P*<0.01).

### Isolation-by-distance

**Figure 5:**
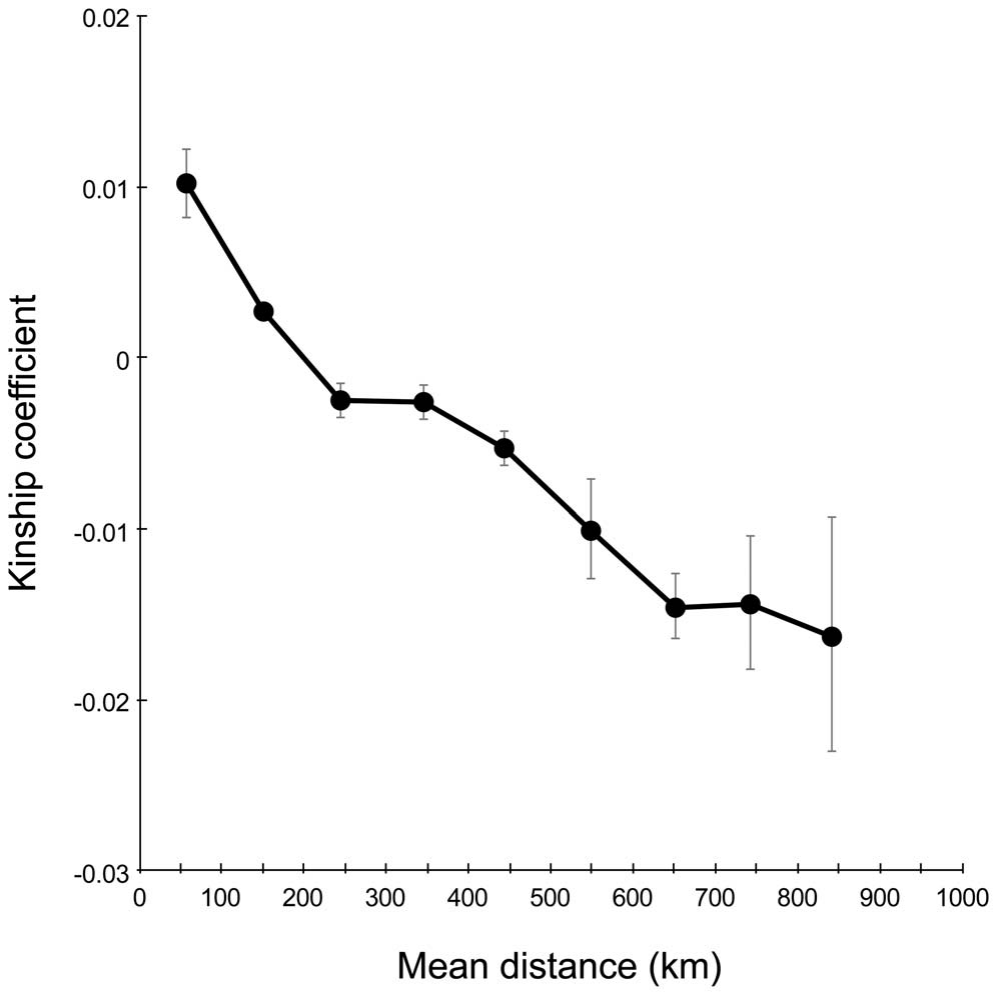
Correlation between geographical distance and kinship of the brown bears in Finland and Russian Karelia. Samples were collected from 2006 to 2010 (*N* = 346) and IBD was analyzed with the program Spagedi 1.3 (Hardy and Vekemans 2002). All nine distance classes differ significantly (*P*<0.001) from the mean kinship of the population.

We detected a significant, negative relationship (*P*<0.001) between kinship and spatial distance between pairs of individuals sampled continuously in Finland and Russian Karelia, providing evidence of an influence of IBD on the degree of genetic structuring. All distance classes showed significant deviation of kinship from the population mean (Figure 5).

### Gene Flow

**Figure 6:**
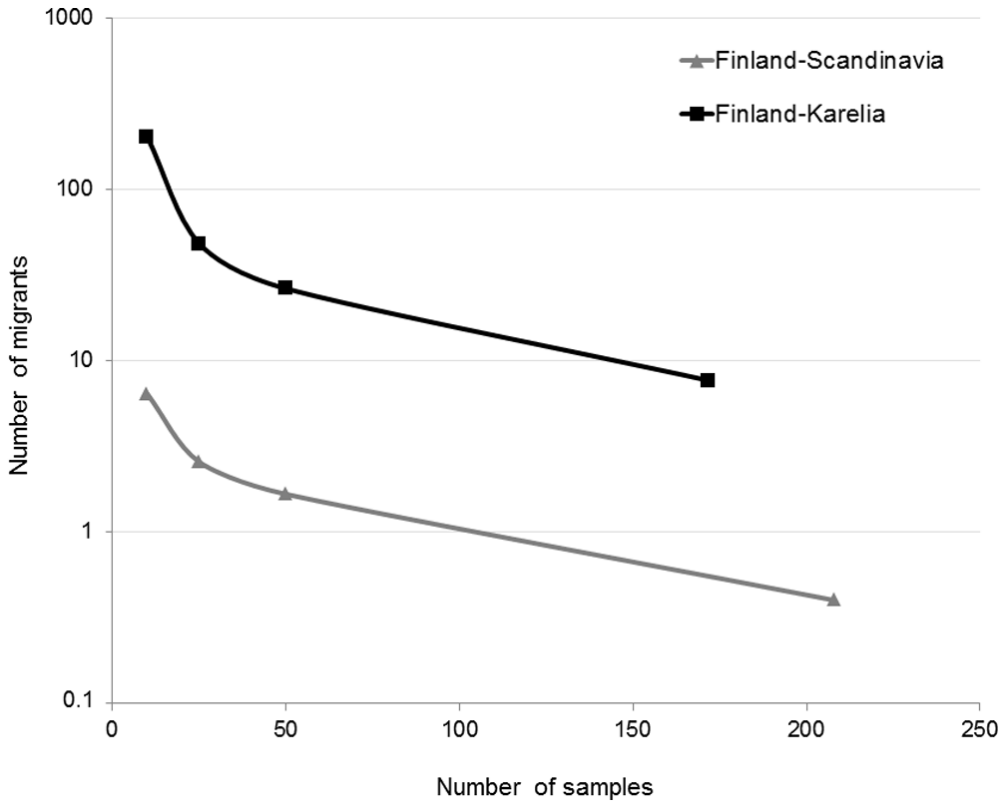
The estimated number of effective migrants per generation (*Nm*). Estimated migrants between Finland and Russian Karelia (black squares) as well as between Finland and Scandinavia (grey triangles) plotted against sample size (see Table S2).

The estimated effective number of migrants was much higher between Finland and Russian Karelia than between Finland and Scandinavia (Figure 6, Table S2). Similar results were found using the software Geneclass 2, which detected 18 migrants between Finland and Russian Karelia. Out of these, 15 bears were identified as first generation migrants from Russia into Finland. In comparison only three individuals were identified as migrants from Finland into Russia. Between Scandinavia and Finland 8 individuals were detected as migrants. All of them were sampled in Finland and originated from Scandinavia.

### Genetic Diversity

Mean observed and expected heterozygosity in Finland were higher in the northern cluster than in the southern one. The northern cluster also showed higher number of alleles per locus than the southern one (Table 1). One locus (G10B) showed deviation from HWE within the southern population, due to excess of heterozygotes. This locus as well as locus Mu05 showed significant, negative values of *F_IS_* (Table 1).

**Table 1:** Genetic diversity of the Finnish brown bear population.

Brown bears in Finland 2006–2010 - Genetic diversity												
	North *(N = 164)*				South *(N = 122)*				North and south (*N = 286)*			
Locus	*A*	*H_E_*	*H_O_*	*F_IS_*	*A*	*H_E_*	*H_O_*	*F_IS_*	*A*	*H_E_*	*H_O_*	*F_IS_*
MU05	10	0.82	0.82	−0.001	9	0.76	0.82	−0.086*	10	0.81	0.82	−0.017
MU09	13	0.89	0.90	−0.014	9	0.86	0.84	0.014	13	0.88	0.88	**0.006**
MU10	10	0.82	0.84	−0.021	9	0.75	0.78	−0.037	10	0.81	0.81	−0.007
MU15	7	0.78	0.76	0.019	7	0.81	0.85	−0.047	7	0.80	0.80	0.001
MU23	13	0.89	0.84	0.050	11	0.81	0.80	0.013	13	0.87	0.83	0.056*
MU50	10	0.76	0.76	0.008	9	0.58	0.56	0.046	10	0.70	0.67	0.040
MU51	9	0.82	0.84	−0.026	7	0.75	0.77	−0.032	9	0.81	0.81	−0.002
MU59	16	0.90	0.91	−0.014	12	0.87	0.83	0.048	16	0.90	0.88	0.021
G1A	10	0.83	0.81	0.031	9	0.82	0.82	0.004	10	0.83	0.81	0.027
G1D	9	0.86	0.87	−0.019	8	0.82	0.79	0.038	9	0.85	0.84	0.012
G10B	12	0.87	0.88	−0.023	8	0.81	0.81	−0.076*	12	0.84	0.88	−0.040
G10L	11	0.77	0.76	0.004	8	0.73	0.75	−0.027	11	0.75	0.76	−0.006
Mean	10.8	0.83	0.83	−0.001	8.8	0.78	0.79	−0.012	10.8	0.82	0.82	**0.008**
* P<0.05												

Expected, (*H_E_*) and observed (*H_O_*) heterozygosities, number of different alleles (*A*) and inbreeding values (*F_IS_*) calculated for the 12 short tandem repeats in the Finnish brown bear population of 286 individuals, sampled from 2006 to 2010. Significant *F_IS_* values are marked with *. Loci deviating significantly from Hardy-Weinberg equilibrium after Bonferroni correction are highlighted by bold *F_IS_* values.

When all genotypes from Finland were pooled, it resulted in the whole population deviating from HWE. One locus (Mu09) deviated as well and showed an elevated, albeit low, positive value of *F_IS_* due to excess of homozygotes (Table 1). This overall deviation from HWE may be most probably caused by the Wahlund effect, by pooling samples from two different genetic clusters into one. *F_IS_* at locus Mu23 was elevated and significant (Table 1).

We found significant linkage disequilibrium (*P*<0.01) after sequential Bonferroni correction in 40 out of 66 marker pairs. Notable is that out of these, 29 pairs were solely found in the Scandinavian cluster (Västerbotten and Troms). Significant LD found was not consistent across all samples and all genetic clusters identified.

### Population Bottlenecks

We detected a genetic bottleneck (*P* = 0.034) for the genetic cluster identified in Scandinavia (Västerbotten and Troms). This cluster showed also the lowest value for the Garza-Williamson index with *M* = 0.64, which is just below *M*_crit_ of *M*<0.68 proposed by Garza and Williamson [58] to suggest the occurrence of a genetic bottleneck in the past.

## Discussion

We have tested the hypothesis that the bear population of Russian Karelia has acted as a source population during the recovery of the Finnish and Scandinavian bear populations. We applied continuous sampling corresponding to the estimated distribution of bears in the area and covered all possible migration routes. Our results showed that the brown bear population in Finland and Russian Karelia consists of two clusters, a northern and a southern one. The clusters showed substantial geographical overlap and the genetic differentiation between them was modest, suggesting a high degree of admixture. Migration analyses supported these findings and showed that gene flow between Finland and Russian Karelia was high, especially in the east-west direction. In comparison, gene flow between Finland and Scandinavia appeared to be restricted, and was found to be absent from the east towards Scandinavia.

The structuring of the Finnish bear population [30] was not as expected, showing a substantial degree of overlap between the clusters. This is most probably caused by the strong influence of IBD on this population. In the previous study by Tammeleht et al. [30], 70 Finnish bear samples were reported to show a pairwise *F_ST_* between two clusters of *F_ST_* = 0.067. This result is considerably higher than in our study and may be an effect of non-continuous sampling [59]. However, it may also be explained in part by invoking the history of the brown bears in the country. With the recent demographic bottleneck of the Finnish bear population in mind, it may be possible that the two clusters were once a single population or both populations may have been connected better in the past and might have been divided during time of persecution. According to historical records, the brown bear was virtually extinct from most parts of Finland, with exception of the area south-east and in the north, near the border to Russia [11]. During the time of extensive persecution the number of individuals plummeted and the population may have become subdivided. The demographic recovery might have connected the clusters again. If that was the case, the differentiation between the two populations was probably never high. The significant, albeit low *F_ST_* values could be explained by such a scenario [3]. Perhaps the northern and the southern cluster identified represent two lineages of recolonization of Finland during the last decades: one lineage from the south-east and another from north-east. The occurrence of historic migration events has been strongly indicated by results of analyses using the mitochondrial genome [32]. However, recent information and data from areas further east remain vague and verification therefore is not possible at this point.

Noticeable is the high number of bears which could not be assigned unambiguously to any of the identified clusters when genetic structure was analyzed on an intermediate scale (Finland and Russian Karelia). The ambiguously assigned individuals were mainly from the admixture zone in the southern part of Finland along the border to Russia (and in Russian Karelia) and raise the question on their origin. However, note that for the cluster assignment analysis on the large scale (incl. Scandinavia), the number of ambiguously assigned individuals was rather low (3.5%). Hence, bears that could not be assigned unambiguously on the intermediate scale were assigned almost completely when compared to a more distant population (i.e. Scandinavia). We believe that these results suggest the existence of a continuous bear population structured by IBD in Finland and Russian Karelia. Consequently, we interpret the low membership coefficients as a likely result of admixture between subpopulations. This might have led to difficulties in clearly assigning individual genotypes to one of the identified clusters during the analyses. However, influence of other bear populations to the east and south, e.g. towards St. Petersburg and further south to Estonia [30] may also be possible. If the bear populations further east or south share indeed the same history of persecution, their recovery and expansion may explain the gradual increase of immigrating individuals from other populations into Finland.

No genetic bottleneck was detected for the bears in Finland and Russian Karelia, as previously indicated for a small part of the population [28], leading to the assumption, that a sufficient number of individuals may have survived during the time of the demographic bottleneck and/or the bottleneck was very short in time; too short to lead to a substantial loss in genetic material. It has been reported earlier, that Russian border fences located along the Finnish-Russian border may prevent or affect wolves roaming in east-west direction [60]. Our results showed that the gene flow across the Finnish-Russian border has been sufficient and that those fences may not constitute a serious obstacle for brown bears.

Connectivity of the Finnish and Russian Karelian brown bears with populations in the west towards Scandinavia seemed more restricted, as our previous study has indicated [25]. All detected first generation migrants in the north were identified as individuals originating from the Scandinavian population, which migrated towards east, into Finland, pointing to unidirectional gene flow. The Scandinavian bear population has its main distribution in Sweden with outliers into Norway. Approximately 30 years after the recovery started, the bears in Sweden were divided into three genetic clusters, which corresponded to areas with high concentration of females [29]. These areas are assumed to represent historic relict areas, in which a few bears have survived the time of intensive hunt [29], [61], [62]. Despite of a genetic bottleneck, the Scandinavian bears showed relatively high levels of heterozygosity (*H_O_* = 0.66) [31], although considerable smaller than found in Finland and Russia in this study. This remarkable mismatch may be the result of the extreme differences in gene flow from Russia. The Finnish bear population is the only connection of the Scandinavian bear population to the Russian one. Although the Finnish and the Scandinavian populations both started off their recovery from being hunted down to near extinction in most parts, their mechanisms of recovery must have been quite different and this is reflected in today’s genetic composition. Our results show that the Finnish population probably has always experienced gene flow from Russia in comparison to the Scandinavian bear population, which recovered without substantial support from other populations.

We propose that future studies should analyze historical samples to elucidate the history of the brown bears in Finland, Russian Karelia and Scandinavia during the time of persecution and initial phases of recovery. Further, analyses on recent migration should be monitored and focus more intensively on bears in different regions by applying noninvasive genetic sampling and estimation of capture-mark-recapture probabilities. This can result in feasible estimations on possible demographical changes, such as reproduction and turn-over rates as well as the ratio between effective and census population sizes.

## Supporting Information

Figure S1 **Bayesian clustering results of Northern European bears with the program Structure (Pritchard et al. 2000).** Samples were collected from 2006 to 2010. (a) Finnish samples only; (b) Finnish and Russian Karelian brown bear samples together as well as (c) brown bear samples from all over the sampling range of northern Scandinavia, Finland and north western Russia of the. Presented are the mean likelihoods *L(K)* and standard deviations for *K* = 1 to 10 clusters over 10 independent runs (1,000,000 iterations and 100,000 burn-in) and the estimate of *ΔK* using the approach described by Evanno et al. (2006). Graphs were plotted using the web based analysis Structure Harvester (Earl and vonHoldt 2012).
(TIF)

Figure S2 **Bayesian clustering results of the Northern European bears with Structure (Pritchard et al. 2000).** Bar plots showing the assignment probabilities for each bear to the identified clusters from *K* = 2 to 5 when only samples from Finland were analyzed (a) and samples from Finland and Russian Karelia pooled together (b). Individuals are arranged by latitude from north (left) to south (right). Bar plots for *K* = 2 to 5 when all data (Finland, Russian Karelia and Scandinavia) is analyzed together (c). Notable is the increase of unassigned individuals (*q*<0.7) with increasing *K*. For Finland, the number for *K* = 2 was 60 (20.98%), while for *K* = 3 to 5 the number increased from 103 (36.01%) to 206 (72.03%).
(ZIP)

Table S1 **Brown bear genotypes (** ***N*** **= 517) used in this study.**
(XLSX)

Table S2 **The estimated number of effective migrants per generation (** ***Nm*** **).** Number of migrants between Finland and Russian Karelia as well as between Finland and Scandinavia. *Nm* results are shown after correction for different sample sizes of 10, 25 and 50 as well as for the mean sample size.
(XLSX)
