# Supplementary figures and images for: Admixture and Gene Flow from Russia in the Recovering Northern European Brown Bear (Ursus arctos)
Source: PLoS One. 2014 May 19;9(5):e97558. doi: 10.1371/journal.pone.0097558 (PMC4026324; doi:10.1371/journal.pone.0097558)

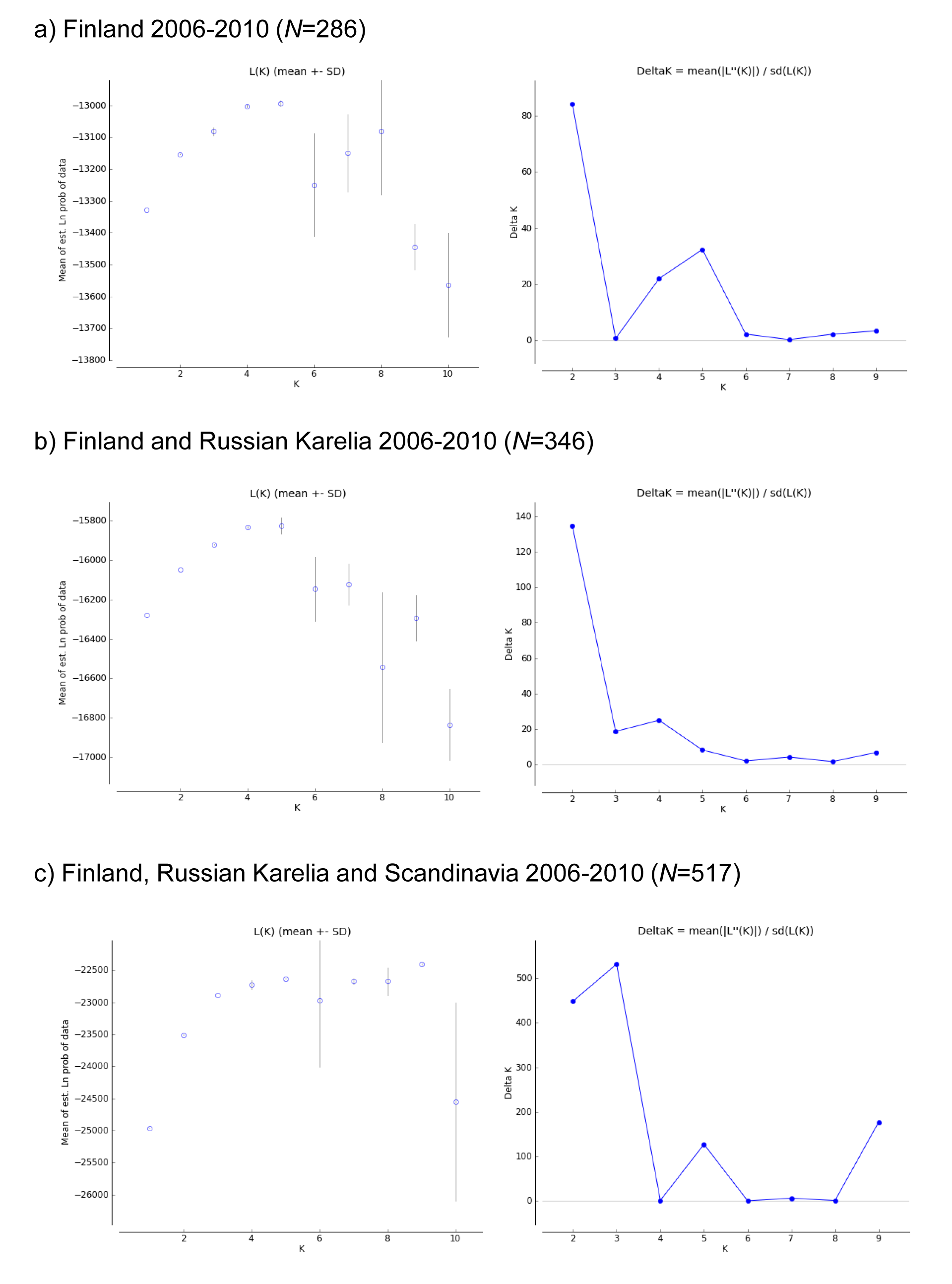

Supplement: Figure S1 — Bayesian clustering results of Northern European bears with the program Structure (Pritchard et al. 2000). Samples were collected from 2006 to 2010. (a) Finnish samples only; (b) Finnish and Russian Karelian brown bear samples together as well as (c) brown bear samples from all over the sampling range of northern Scandinavia, Finland and north western Russia of the. Presented are the mean likelihoods L(K) and standard deviations for K = 1 to 10 clusters over 10 independent runs (1,000,000 iterations and 100,000 burn-in) and the estimate of ΔK using the approach described by Evanno et al. (2006). Graphs were plotted using the web based analysis Structure Harvester (Earl and vonHoldt 2012). (TIF) [file pone.0097558.s001.tif]

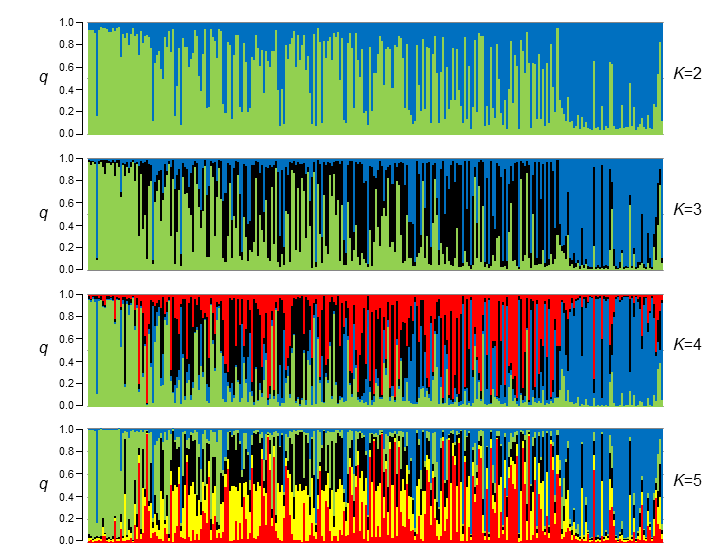

Supplement: Figure S2 — Bayesian clustering results of the Northern European bears with Structure (Pritchard et al. 2000). Bar plots showing the assignment probabilities for each bear to the identified clusters from K = 2 to 5 when only samples from Finland were analyzed (a) and samples from Finland and Russian Karelia pooled together (b). Individuals are arranged by latitude from north (left) to south (right). Bar plots for K = 2 to 5 when all data (Finland, Russian Karelia and Scandinavia) is analyzed together (c). Notable is the increase of unassigned individuals (q<0.7) with increasing K. For Finland, the number for K = 2 was 60 (20.98%), while for K = 3 to 5 the number increased from 103 (36.01%) to 206 (72.03%). (ZIP) [file pone.0097558.s002.zip › S2a.tif]

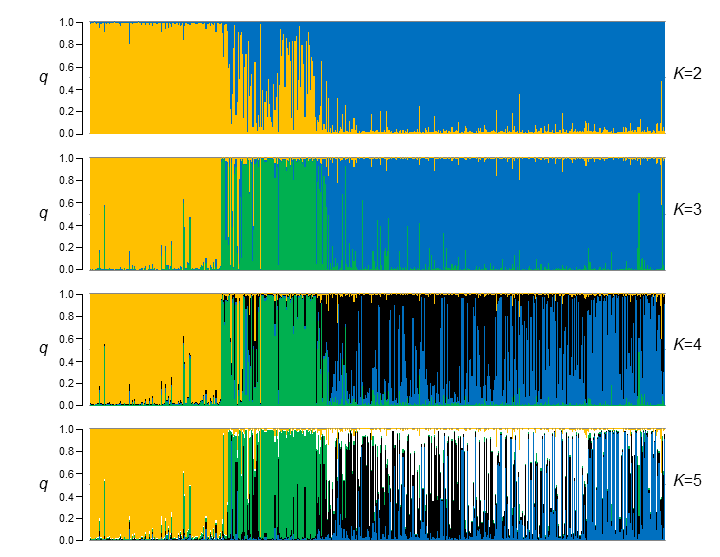

Supplement: Figure S2 — Bayesian clustering results of the Northern European bears with Structure (Pritchard et al. 2000). Bar plots showing the assignment probabilities for each bear to the identified clusters from K = 2 to 5 when only samples from Finland were analyzed (a) and samples from Finland and Russian Karelia pooled together (b). Individuals are arranged by latitude from north (left) to south (right). Bar plots for K = 2 to 5 when all data (Finland, Russian Karelia and Scandinavia) is analyzed together (c). Notable is the increase of unassigned individuals (q<0.7) with increasing K. For Finland, the number for K = 2 was 60 (20.98%), while for K = 3 to 5 the number increased from 103 (36.01%) to 206 (72.03%). (ZIP) [file pone.0097558.s002.zip › S2c.tif]

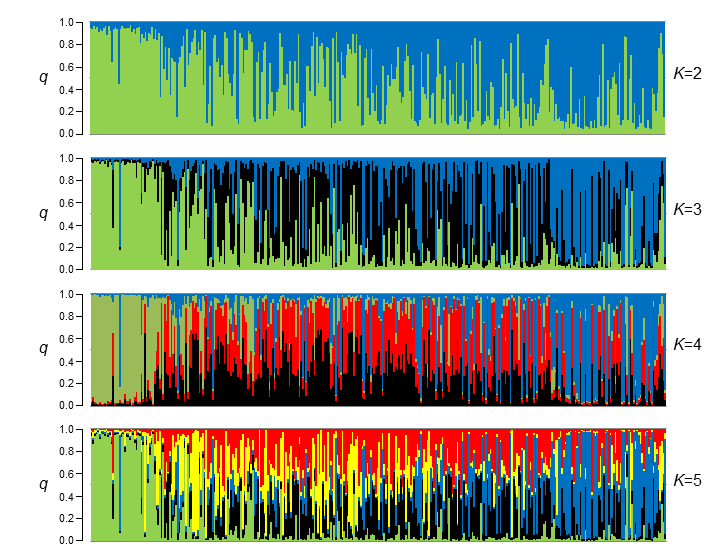

Supplement: Figure S2 — Bayesian clustering results of the Northern European bears with Structure (Pritchard et al. 2000). Bar plots showing the assignment probabilities for each bear to the identified clusters from K = 2 to 5 when only samples from Finland were analyzed (a) and samples from Finland and Russian Karelia pooled together (b). Individuals are arranged by latitude from north (left) to south (right). Bar plots for K = 2 to 5 when all data (Finland, Russian Karelia and Scandinavia) is analyzed together (c). Notable is the increase of unassigned individuals (q<0.7) with increasing K. For Finland, the number for K = 2 was 60 (20.98%), while for K = 3 to 5 the number increased from 103 (36.01%) to 206 (72.03%). (ZIP) [file pone.0097558.s002.zip › S2b.tif]
